# Supplementary material for: Maternal cytomegalovirus infection and delayed language development in children at 3 years of age–a nested case-control study in a large population-based pregnancy cohort
Source: PLoS One. 2022 Dec 1;17(12):e0278623. doi: 10.1371/journal.pone.0278623 (PMC9714838; doi:10.1371/journal.pone.0278623)
Supplement: Attachment — Submitted filename: Comment.docx [file pone.0278623.s001.docx]

**Maternal cytomegalovirus infection and delayed language development in children at 3 years of age – a nested case-control study in a large population-based pregnancy cohort**

Reviewer comment:

Reviewer name: Abel Gedefaw ( MD,MPH)

Associate professor of OBY/GYN

Hawassa University, Ethiopia

General comment

- The topic is so relevance and timely to improve maternal and child health
- The paper is well written , but need to address points raised in detail below

**Abstract**:

**Conclusion**: Only the odds of CMV seroprevalence positivity, which had a significant association, are included in the conclusion part. The non-significant association of seroconversion odds should also be addressed in the conclusion.

**Introduction:**

Only one study, a Dutch study, mentioned the effects of CMV infection and language development. It is good to include more studies on CMV infection and language development to justify the importance of the study.

Other risk factors for delay language development like other infections could be mentioned briefly and the importance of CMV infection study can be elaborate.

**Materials and methods**

i. **Study ( Cohort ) population description**
1. Briefly describe the objective of the cohort for a better understanding of the paper
2. It is good to describe briefly what was done or measured for the cohort population related to CMV infection and outcome despite it was mentioned in other papers. The only measurement stated in the manuscript was blood sample was taken for seroconversion and 2^nd^ trimester CMV positivity analysis. Despite we assumed nearly half of the primary infection end up with congenital infection based on previous data, it is good to include the following if it was measured.

- Diagnosis of congenital CMV infection
- Diagnosis of symptomatic CMV infection at birth
- Whether there were follow-ups of infants born with CMV converted mothers for short and long-term sequel

ii. **Selection of cases need clarification.**

It is stated only as 300 cases of delayed language development. Among the 95,200 pregnant women cohort

- How many children did have delayed language development
- How did you select the 300 cases? How many cases were excluded from analysis due to other exclusion criteria ( Multiple pregnancies, chromosomal disorders, other study participation )

**Other variables:**

Only educational status mentioned. But in the result part, age, parity, gestational age,BMI, mentioned.

**Statistical analysis**

It is good to add the reference for sample size calculation

**Result**:

Despite it is case –control study, it is good to include incidence data related to CMV infection among the cohort population since it is a national and large sample cohort

- What is the incidence of CMV positivity at the 2^nd^ trimester (could be primary infection in the first trimester or reactivation of latent infection) and seroconversion rate at birth?
- Among the total seroconverted women

How many of them had been diagnosed with congenital CMV infection?

How many of them were symptomatic at birth for CMV infections?

If there was no congenital infection diagnosis done, it should be stated in the discussion part regard to the clinical benefit of only maternal seroconversion at birth without a diagnosis of congenital infection. If we decrease the cases by half considering only half of the primary infection will have a congenital infection, do the risk of delayed language development association still persist

- Table 3: add the P-Value for each variable to know the presence of association
- Table 4: it is good to show the confounding variables crud and adjusted odd ratio too( parity, age, education, hearing impairment, etc for delayed language development

**Discussion**

It states “ Seropositivity and seroconversion rates in our study are comparable to other studies among pregnant women”. This is difficult to conclude in case-control study. As mentioned above, we need to know the cohort result.

It is good to discuss the limitation of

1. Do we exclude other congenital infections such as syphilis, toxoplasmosis, and rubella based on maternal and infants’ serological testing? How do we control the confounding effect of other infections? Despite it is a case-control study and we need to mention the importance of excluding other infections in the limitation part.
2. Ascertainment of congenital infection ( Seroconversion Vs congenital infection and language development delay )

**Conclusion**: see above in the abstract
