# Supplementary material for: Maternal cytomegalovirus infection and delayed language development in children at 3 years of age–a nested case-control study in a large population-based pregnancy cohort
Source: PLoS One. 2022 Dec 1;17(12):e0278623. doi: 10.1371/journal.pone.0278623 (PMC9714838; doi:10.1371/journal.pone.0278623)
Supplement: Attachment — Submitted filename: PONE-D-22-17172R2_Response_letter.docx [file pone.0278623.s003.docx]

**Reviewer response letter**

Ref. No.: PONE-D-22-17172R1

Title: Maternal cytomegalovirus infection and delayed language development in children at 3 years of age – a nested case-control study in a large population-based pregnancy cohort.

We would again like to thank the reviewers and the editor for the time and effort that has been put into assessing the previous version of the manuscript. Please find our point-by-point responses to the comments below.

Review Comments to the Author

Reviewer #1: The manuscript became clearer and improved.
I recognized that the authors main interest is the association between maternal CMV infection defined as serological positive or seroconversion and delayed language development regardless of the co-existence of hearing impairment.
The outcome is defined as moderate or severe problem of in ASQ question 18 (speech delay) AND simultaneous score <=40 in ASQ question 21 (cognitive developmental delay).

Please see explanation below about question 18 and 21.

Authors answered all the questions, but I would like to clarify the major issue on the statistical analysis again.
1. If the authors think that hearing impairment is a mediator, they should not include the hearing impairment in the multivariate logistic regression in (Table 5 and 6). Just use model 1 and model 2.
2. If the authors think that hearing impairment is a confounder, they can keep the hearing impairment in the multivariate logistic regression and probably model 2 is just CMV IgG+ or seroconversion and hearing impairment, which is the authors' main interest. It is up to them to include other variables in model 3.

So please change the wording from mediator to confounder if authors would like to keep the current analysis. (line 198)
Alsos no need to mention mediator analysis in lines 269-271 if authors are going to take hearing impairment as a confounder.

Response to poins 1 and 2:

The reviewer is correct that our main interest of the study is to ascertain the effect of CMV on speech development, but because the role of hearing impairment is unclear we have tried to account for this in our analyses and how we present our results. We do not think it is wise to commit to one of the two possibilities and pretend the other is irrelevant. We would also like to point out that if hearing impairment is indeed a mediator, it still makes sense to include an analysis where hearing impairment is a predictor. In the mediator case this would make the coefficients for CMV interpretable as direct effects. Hence, the model with hearing impairment included is relevant for both the mediator and confounder scenarios.

We revised Results and Discussion to clarify this issue. Please see lines 153-155, 185, 205 and 251 – 254.

I have one question in the definition of the case. ASQ question 21 is detecting the mental delay. I think the authors would like to remove this question 21 from the case definition as the language development delay without cognitive disorder was excluded in the current definition.

The two questions are both exploring the language development in children. They were not developed to detect mental delay. Question 18 describes the speech performance and question 21 the child’s ability regarding language comprehension. The two questions are used in combination to select cases in the most appropriate way to ensure a consolidated group of children with language problems. A validation was conducted initially to assess the questionnaire’s ability to detect language development by comparing the parent`s response to these questions to a concomitant psychological examination of the child at 3 years showing high correlation. We have included a paragraph in the re-revised manuscript about this comparison and a new reference.

The selection of cases in this study is based on both Question 18 and 21 to obtain a consolidated group of children with language problems. This means that both questions are required for the analyses and Question 21 cannot be omitted from this study.

I also suggest that column of case and controls in table3 and table 4 should be combined as a result of univariate logistic regression analysis. CMV 1gG seropositive and seronegative should be in an independent table before Table 3.
Table 3 should include the age as a potential confounder.

In the revised Table 3 we have included a column with p-values for comparing all the variables between cases and controls. We have included a new footnote with an explanation to this variable. This table also compares mean age between cases and controls as well as between CMV seropositives and seronegatives. No difference was found comparing age between the groups.

Results section also includes Table 4 with a comparison of CMV IgG antibody status between cases and controls.

In statistical analysis lines 147-149, it should be "Odds ratios with 95% confidence intervals were calculated with univariate logistic regression models, and adjusted ORs were calculated multivariate logistic regression adjusting for hearing impairment (if hearing impairment is recognized as a confounder), age, parity and maternal educational status." IF sensitivity analysis is going to be conducted, please mention that in the statistic section.

We have revised accordingly in the Statistical analyses.

Line 250 authors said that age did not influence the risk of being IgG positive in our study. This result was not mentioned in the result section. I suggest including the age in table 3 or new table.

We thank the reviewer for the constructed comment and we understand that the sentence could be misunderstood. We have revised this sentence and included the reference to the previous study.

It is also referred to age in the beginning of the result section, stating that age was similar for CMV seropositive status compared to seronegative. Table 3, already includes age as a variable.

Conclusion said that "this population based study showed a higher risk of delayed language development ..." But I suggest "showed" is a bit strong conclusion while this study did not show the association between them. The expression may be,, "this study supported the previous study finding of ......."

We thank the reviewer for suggesting a revised Conclusion, which we have implemented.
